# The mediation and moderation effect of social support on the relationship between opioid misuse and suicide attempts among native American youth in New Mexico: 2009-2019 Youth Risk Resiliency Survey (NM-YRRS)

**DOI:** 10.1186/s12888-022-03900-8

**Published:** 2022-04-05

**Authors:** Daniel Opoku Agyemang, Erin Fanning Madden, Kevin English, Kamilla L. Venner, Rod Handy, Tejinder Pal Singh, Fares Qeadan

**Affiliations:** 1 Department of Family and Preventive Medicine, University of Utah School of Medicine, Salt Lake City, USA; 2 Department of Family Medicine and Public Health Sciences, Wayne State University, Detroit, USA; 3 Albuquerque Area Southwest Tribal Epidemiology Center, Santa Fe, NM USA; 4 Department of Psychology, Center on Alcohol, Substance use, And Addiction (CASAA), University of New Mexico, Albuquerque, USA; 5 Parkinson School of Health Sciences and Public Health, Loyola University Chicago, Maywood, IL, US United States

## Abstract

**Background:**

Suicide attempt and opioid misuse continue to be major behavioral health challenges among American Indians and Alaska Natives (AI/AN). The aim of the study is to evaluate the mediating and moderating role that social support (SS) plays in their association among AI/AN high-school students in New Mexico (NM).

**Methods:**

An aggregated NM Youth Resiliency and Risk Survey (NM-YRRS, 2009-2019: odd years) dataset was used. Multivariable logistic regression modeling and mediation analysis were conducted while adjusting for confounding variables.

**Results:**

Overall, 12.0 and 14.0% of AI/AN students reported opioid misuse and suicide attempt, respectively. The adjusted odds ratio of suicide attempt in students with high SS relative to low SS who misused opioids was 0.43 (*p*-value = 0.007). The effect of high SS relative to low SS among males who misused opioids was more pronounced (AOR = 0.24, *p*-value < 0.0001) compared to females (AOR = 0.43, *p*-value = 0.007). Relative to low SS, high SS was protective for suicide attempt among AI/AN students who misused opioids and attended school in off-reservation (AOR = 0.42, *p*-value = 0.012) communities, rural communities (AOR = 0.44, *p* = 0.040), and in communities that are both rural and off-reservation (AOR = 0.39, *p* = 0.035). Overall, 23.64, and 41.05% of the association between opioid misuse, and suicide attempt was mediated and moderated by SS, respectively. The mediation effect of SS was lowest for rural, on-reservation schools.

**Conclusion:**

More resources need to be allocated to rural on-reservation schools to enhance social support. The study highlights key insights into the significant role SS plays in promoting health and mitigating the association between opioid misuse and suicide attempt.

## Introduction

American Indian and Alaska Native (AI/AN) populations have disproportionately higher suicide rates than the overall U.S. population [1, 2]. In the United States (US), suicide deaths occur mainly in midlife [3]; however, AI/AN populations experience the highest suicide rates during adolescence and young adulthood [4–6]. Suicide is the eighth leading cause of death among AI/AN across all ages and the second leading cause of death among those ages 10 – 34 in the US [7]. A national Youth Risk and Behavioral Survey (YRBS) from 1999 to 2015 reported that of all racial groups, AI/AN adolescents had the highest prevalence of lifetime and current use of every substance studied except for heroin, and reported the highest prevalence of attempted suicide, which was almost three times greater than non-Hispanic white adolescents [8]. New Mexico (NM) is among the top four states in the country for suicide deaths [9]. Suicide death rates in NM have been consistently higher than the national average [10]. In particular, the age-adjusted suicide death rate in NM is 23.2 per 100,000 compared to the national average, which was 14.0 per 100,000 in 2017 [10]. Although NM has seen an increase in suicide death in all age groups, from 1999 to 2017, the most significant increase (50%) was among youth 10 – 24 years, from 15.5 to 23.3 deaths per 100,000 [11]. In 2017, 14 and 13% of high school and middle school students in NM reported attempted suicide, respectively [12].

Research has shown that people who use opioids are thirteen times more likely to have suicide ideation [13]. A study among young adult patients admitted for suicide attempt revealed that they were more likely than the healthy control group to have misused opioids in the past year prior to hospitalization after controlling for past suicide attempt [14]. A study of high school students in the U.S. also revealed that adolescents who reported a history of heroin use had the strongest association with suicide attempts compared to peers who never used heroin [15]. In 2017, 14% of US adolescents reported misusing opioids. A more recent study from 2020 reported that adolescents who misuse opioids were 4.9 times more likely to have ever attempted suicide [16].

The opioid overdose death rate among AI/AN rose between 1999 to 2016 from 2.9 per 100,000 to 13.9 per 100,000 [17]. Other risk factors for AI/AN youth opioid misuse include feeling hopeless and sadness [18], and reporting anxiety and depression [19, 20]. Structural factors including years of injustice [21], historical colonization, and other structural determinants of health [22] have marginalized AI/AN communities in ways that affect upstream risk and protective factors and downstream health outcomes. Consequently, AI/AN youth face stark inequities to healthy development [23], and rank higher in health disparities relative to other racial and ethnic groups in the US [24]. Poverty, institutional racism, discrimination, disparities in health care access, health care delivery, and historical trauma play a significant role in the health status of AI/AN [25], including increasing risk for cancer, substance use disorders, obesity, and heart disease compared to the general population [26].

Household socioeconomic status and community traits may play a role in youth behavioral health as well. One measure of socioeconomic status is maternal education, which previous research suggests plays a critical role in suicide attempt and opioid misuse [27]. There is an increased risk for emotional and behavioral disorders among children whose mothers have low educational attainment [28]. A 26-year retrospective review of youth suicide in New Mexico revealed that if left untreated, these emotional and behavioral disorders could lead to increased risk of suicide attempt [29]. Living in a rural community is also associated with increased risks for drug use [30], as well as suicide and self-inflicted injuries [31, 32].

Among AI/AN youth, social support has been found to be a protective factor associated with decreased odds of opioid misuse and suicide attempt [12, 27, 33]. Social support is operationalized in several different ways. For the sake of this study, social support is conceptualized in terms of interpersonal and structural engagements that provide functional support for AI/AN youth. Some social network factors that can protect AI/AN youth behavioral health include a sense of belonging to one’s culture, a strong tribal or spiritual bond, an opportunity to discuss challenges with family or friends, family connectedness, and positive emotional health [34, 35]. Other protective factors include college aspirations and good academic performance [36], positive self-image [35], feeling cared about by adults [37], community factors like participation in sports and clubs [35], and enculturation [38, 39]. In addition, several studies of adolescents reported strong familial attachment [40–42], school connectedness [40, 43], and community characteristics like opportunities to serve and community support groups [44] are associated with lower probability of suicide attempt. However, some social network factors like low parental involvement in the daily activities of the youth, engaging with peers who use illicit substances, and less familial disapproval of substance use can also be risk factors for opioid misuse [19, 45, 46].

The role of social network factors in the relationship between opioid misuse and suicide attempt among AI/AN middle and high school students remains unclear. The extant literature shows that social and familial support are relevant to suicide and substance use for AI/AN adolescents [17, 47–49]. And due to the importance of family and social support, some tribes have integrated cultural support structures into clinical and behavioral therapy [17], and utilized strong family and community connections to mitigate risky health behaviors [33, 50, 51]. Yet, the extent to which social networks may operate as a protective factor modifying the association between opioid misuse and suicide attempt among AI/AN youth is notably lacking. The current study aims to estimate the mediation and moderation effect of social support on the relationship between opioid misuse and suicide attempt among AI/AN youth in New Mexico in order to better understand the role of this key protective factor in AI/AN behavioral health.

## Methods

### Data source and design

This is a repeated cross-sectional design with deidentified respondents who were not able to be matched between survey years. Aggregated data from 6 years (2009, 2011, 2013, 2015, 2017, and 2019) of the high school New Mexico Youth Risk and Resiliency Survey (NM-YRRS) were obtained from the New Mexico Department of Health (NM-DOH). The NM-YRRS uses a two-stage cluster sampling design to produce a representative sample of high school students in grades 9-12 [52]. However, Albuquerque Area Southwest Tribal Epidemiology Center (AASTEC) assisted in oversampling AI/AN youth to provide a more robust and representative sample of AI/AN students [52]. This study was determined to be exempt by the University of Utah Institutional Review Board (IRB #137165). AASTEC, through NM-DOH, provided the oversampled data for the study after a data-use agreement was signed. A Community Advisory Board (CAB) and AASTEC were instrumental in providing insights into the results and findings of this study. The CAB reviewed the major findings in a meeting and provided feedback on interpretations of the results presented in this study.

### Measures

For consistency, only variables asked in all survey years were included in the analysis. The primary outcome of interest was a self-reported indicator (binary 0 or 1) for one or more suicide attempts in the past year. Suicide attempt was obtained by asking the question, “*During the past 12 months, how many times did you actually attempt suicide*,” with possible responses of zero through six or more times.

The primary exposure variable was opioid misuse, which was obtained through combining the following two questions, “*During your lifetime, how many times have you used heroin (also called smack, junk, or China White?*” and “*During the past 30 days, how many times did you use a painkiller to get high, like Vicodin, OxyContin (also Oxy or OC), or Percocet (also called Percs)?*” Combining these two questions gave us the ability to explore the effects of overall misuse involving any non-prescribed opioid [53].

The mediator/moderator variable of interest was social support. The social support construct was developed using eight individual survey questions described in Table 1. The responses to these questions were categorized into “Not true at all or A little true,” and “Pretty much true or Very much true.” A response of “Pretty much true or Very much true” was coded as 1, indicative of positive social support, whereas a response of “Not true at all or A little true” was coded as 0, indicative of negative social support. The final composite scores were then categorized into three levels of social support. A score of three or fewer was classified as “Low Social Support,” a score of four to six was classified as “Moderate Social Support,” and a score of seven and above was categorized as “High Social Support” [27]. Rurality and reservation status were determined by high school location. Specifically, rural and urban areas were determined by population density based on predefined parameters set by the US Census Bureau in 2010, where urban areas have 50,000 or more people and urban clusters have at least 2500 but less than 50,000 people [54]. If a high school was in a census-defined urban area, it was categorized as an urban high school, and if a school was in a census-defined rural area, it was classified as a rural high school. Schools that were adjacent to a reservation or off-reservation were classified as off-reservation schools and schools on tribal lands were classified as on-reservation schools.

**Table 1:** Individual social support survey questions

1. Parent or adult at home is interested in my school work?	
2. Parent or adult at home believes I will be a success?	
3. Teacher or adult at school listens to me?	
4. Teacher or adult believes I will be a success?	
5. Adult in the community cares about me?	
6. Adult in the community tells me good job?	
7. A friend my own age really cares about me?	
8. When I am not at home, a parent or guardian knows where I am and who I am with?	

Other confounding variables adjusted for in the analyses include age (< 14 years, 15 years, 16 years, and ≥ 17 years), sex (male, female), year (odd years from 2009 to 2019 inclusive), grade (9th, 10th, 11th, and 12th), academic performance (high grades, poor grades), sexual identity (heterosexual/straight, gay/lesbian, bisexual, and questioning), and maternal education (<high school, high school, and college+).

### Data analysis

**Fig. 1:**
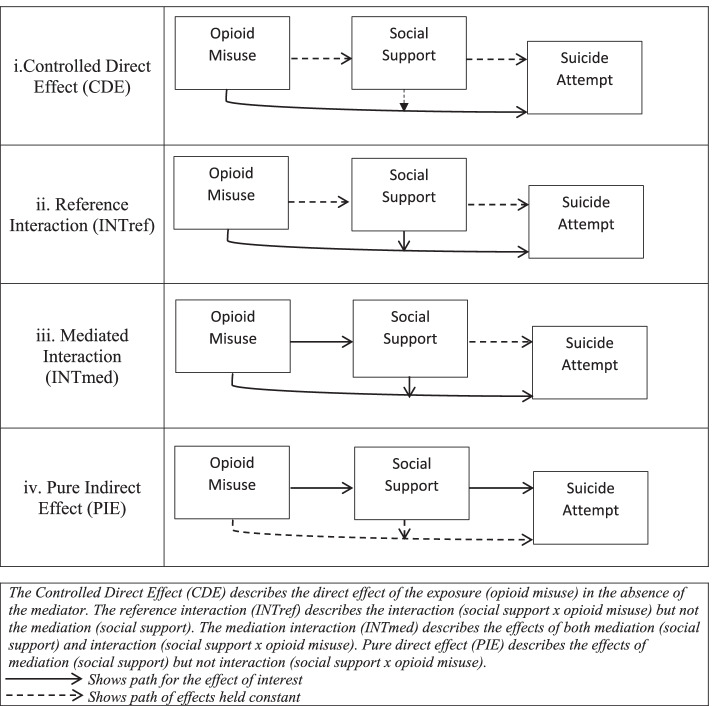
Risk model and the four-way decomposition of mediation and interaction analysis. This figure was inspired from Fig. 1 in Bean et al. [40]

Frequencies and relative frequencies were used for descriptive statistics while accounting for the complex design of the YRRS survey. Multivariable logistic regression modeling was used to estimate the odds of suicide attempt for those who misused opioids relative to those who did not. Further, mediation analysis was employed to assess the mediation/moderation effect of social support on the association between opioid misuse and suicide attempt. In this mediation analysis, the total effect (TE) is expressed in a four-way decomposition as TE = CDE + INT_ref_ + INT_med_ + PIE where (i) controlled direct effect (CDE) captures the percentage of direct effect of the exposure (opioid misuse) in the absence of the mediator (social support); (ii) reference interaction (INT_ref_) captures the percentage of interaction (social support x opioid misuse) but not mediation (social support); (iii) mediated interaction (INT_med_) captures the percentage of both mediation (social support) and interaction (social support x opioid misuse); and (iv) pure indirect effect (PIE) captures the percentage of mediation (social support), but not interaction (social support x opioid misuse) [55] (Fig. 1). The mediation analysis was then stratified by sex (males and females), reservation status (attending school on- or off-reservation), rurality (attending school in a rural or urban location), and the combination between on/off reservation status and rurality (attending school in rural off-reservation, rural on-reservation, or urban off-reservation areas). All statistical analyses were performed using Stata 16.1 (StataCorp, 2019) and the SAS 9.4 system (SAS Institute Inc., Cary, NC, USA).

## Results

### Sample characteristics

An aggregated total of 19,067 AI/AN students surveyed from 2009 through 2019 were included in the study. Out of the total participants, 12.0% reported opioid misuse. A slight majority of the students were males (50.6%), and about 28.6% were over 17 years old. Throughout the survey years, a majority of the students attended high schools in rural areas (66.0%) and in off-reservation communities (87.0%). Half (50.3%) of the students had mothers who were high school graduates, and 32.0% had college education. AI/AN students who identified as heterosexual/straight comprised 81.1% of the sample, 2.9% identified as gay or lesbian, 11.4% identified as bisexual, and 4.6% identified as questioning (Table 2). A slight majority of the students received high social support (50.9%). Except for rurality of school and sex of students, imbalance was observed in the demographic variables between those who misused opioids and those who did not (Table 2).

**Table 2:** Characteristics of AI/AN youth participants (New Mexico YRRS 2009-2019)

	Total n (%^**1**^)	AI/AN Youth with Opioid Misuse n (%^**2**^)	AI/AN Youth with no Opioid Misuse n (%^**2**^)	*P*-value
Total	19,067 (100%)	2363 (12.0)	16,704 (88.0)	NA
**Mediating variable**				
Social Support (SS)				
Low SS	3258 (17.5)	720 (22.0)	2538 (78.0)	< 0.0001
Moderate SS	5945 (31.6)	874 (14.5)	5071 (85.5)	
High SS	9864 (50.9)	769 (7.3)	9095 (92.7)	
**Control variables**				
Age (years)				
≤ 14	3826 (20.8)	361 (8.7)	3465 (91.3)	0.0001
15	4936 (26.1)	612 (12.8)	4324 (87.2)	
16	4899 (24.5)	640 (12.9)	4259 (87.1)	
≥ 17	5406 (28.6)	750 (13.4)	4656 (86.6)	
Sex				
Female	9807 (49.4)	1202 (11.8)	8605 (88.2)	0.3870
Male	9260 (50.6)	1161 (12.5)	8099 (87.5)	
Grade				
9th	5626 (29.9)	614 (9.8)	5012 (90.2)	0.0039
10th	5053 (26.7)	631 (13.2)	4422 (86.8)	
11th	4511 (23.0)	618 (13.3)	3893 (86.7)	
12th	3762 (20.4)	473 (11.9)	3289 (88.1)	
Academic performance				
High grades (A’s / B’s)	11,577 (69.0)	1106 (9.5)	10,471 (90.5)	< 0.0001
Poor grades (C, D, or F’s)	5143 (31.0)	902 (17.0)	4241 (83.0)	
Maternal education level				
< High School	2349 (18.0)	352 (15.6)	1997 (84.4)	0.0001
High School	7534 (50.0)	864 (10.9)	6670 (89.1)	
≥ College	4437 (32.0)	459 (10.0)	3978 (90.0)	
Sexual identity				
Heterosexual	10,633 (81.1)	869 (8.1)	9764 (91.9)	< 0.0001
Gay/Lesbian	353 (2.9)	107 (29.9)	246 (70.1)	
Bisexual	1493 (11.4)	315 (22.1)	1178 (77.9)	
Questioning	547 (4.6)	96 (22.4)	451 (77.6)	
**Stratifying variables**				
Reservation status of school				
On	4066 (13.0)	484 (10.0)	3582 (90.0)	0.041
Off	14,108 (87.0)	1767 (12.0)	12,341 (88.0)	
Rurality status of school				
Rural	13,782 (66.0)	1727 (13.0)	12,055 (87.0)	0.187
Urban	4392 (34.0)	524 (11.0)	3868 (89.0)	

The study revealed that higher levels of social support were associated with lower levels of opioid misuse (from 22.0 to 14.5% to 7.3% among students reporting low, moderate, and high social support, respectively). In addition, we observed a higher level of opioid misuse was associated with increasing age (8.7% among those age ≤ 14 years to 13.4% among those age ≥ 17 years). AI/AN students attending off-reservation schools had a higher prevalence of opioid misuse than those on-reservation (12.0% vs. 10.0%), whereas those attending rural schools also had a higher prevalence of opioid misuse than those attending urban schools (13.0% vs. 11.0%--Table 2). A striking disproportionality of opioid misuse was detected among AI/AN students who identified as gay/lesbian (29.9%), bisexual (22.1%), and questioning (22.4%) compared to their heterosexual counterparts (8.1%).

1% = column percentage.

2% = row percentage.

### Inferential statistics

In the overall sample using multivariable analysis and controlling for nine potential confounding variables, odds ratios for social support were significant in four models. AI/AN students who reported high social support and no opioid misuse had the lowest odds ratio of suicide attempts (AOR = 0.38; 95% CI: 0.27 – 0.54; *p* < 0.0001) compared to those with low social support. Students reporting high social support who also reported opioid misuse had significantly lower odds of suicide attempt (AOR = 0.43; 95% CI: 0.23 – 0.79; *p* = 0.007) compared to those with low social support (Table 3). Male AI/AN students who reported high social support and opioid misuse had a significantly lower odds ratio for suicide attempt (AOR = 0.24; 95% CI: 0.12 – 0.46; *p* < 0.0001) compared to those with low social support. Likewise, males who reported moderate social support and opioid misuse had a significantly lower odds ratio for suicide attempt (AOR = 0.49; 95% CI: 0.27 – 0.88; *p* = 0.016) compared to those with low social support. In addition, male students who reported high social support and no opioid misuse had an odds ratio of 0.21 (95% CI: 0.13 – 0.33; *p* < 0.0001--Table 3) for suicide attempt compared to those with low social support. Overall, no significant associations were found for students who reported moderate social support and either opioid misuse or no opioid misuse relative to those who reported low social support.

**Table 3:** Adjusted odds ratios for suicide attempt and the interaction effect of Social Support and Opioid Misuse

Overall	AOR	95% CI	*P*-value
High SS vs Low SS among opioid users	0.43	0.23 – 0.79	0.007
High SS vs Low SS among non-opioid users	0.38	0.27 – 0.54	< 0.0001
Moderate SS vs Low SS among opioid users	0.89	0.53 – 1.50	0.662
Moderate SS vs Low SS among Non-opioid users	0.72	0.51 – 1.02	0.066
High SS vs Moderate SS among opioid users	0.48	0.29 – 0.79	0.004
High SS vs Moderate SS among non-opioid users	0.52	0.39 – 0.71	< 0.0001
**Among Females**			
High SS vs Low SS among opioid users	0.43	0.23 – 0.79	0.007
High SS vs Low SS among non-opioid users	0.38	0.27 – 0.53	< 0.0001
Moderate SS vs Low SS among opioid users	0.89	0.53 – 1.50	0.662
Moderate SS vs Low SS among Non-opioid users	0.72	0.51 – 1.02	0.066
High SS vs Moderate SS among opioid users	0.48	0.29 – 0.79	0.004
High SS vs Moderate SS among non-opioid users	0.52	0.39 – 0.71	< 0.0001
**Among Males**			
High SS vs Low SS among opioid users	0.24	0.12 – 0.46	< 0.0001
High SS vs Low SS among non-opioid users	0.21	0.13 – 0.33	< 0.0001
Moderate SS vs Low SS among opioid users	0.49	0.27 – 0.88	0.016
Moderate SS vs Low SS among Non-opioid users	0.40	0.26 – 0.62	< 0.0001
High SS vs Moderate SS among opioid users	0.27	0.15 – 0.46	< 0.0001
High SS vs Moderate SS among non-opioid users	0.29	0.19 – 0.43	< 0.0001
**On Reservation**			
High SS vs Low SS among opioid users	0.63	0.12 – 3.45	0.589
High SS vs Low SS among non-opioid users	0.37	0.20 – 0.68	0.002
Moderate SS vs Low SS among opioid users	0.31	0.07 – 1.38	0.121
Moderate SS vs Low SS among Non-opioid users	0.41	0.20 – 0.86	0.019
High SS vs Moderate SS among opioid users	2.05	0.75 – 5.55	0.155
High SS vs Moderate SS among non-opioid users	0.90	0.52 – 1.56	0.708
**Off Reservation**			
High SS vs Low SS among opioid users	0.42	0.21 – 0.82	0.012
High SS vs Low SS among non-opioid users	0.37	0.25 – 0.56	< 0.0001
Moderate SS vs Low SS among opioid users	1.02	0.59 – 1.78	0.943
Moderate SS vs Low SS among Non-opioid users	0.75	0.50 – 1.12	0.154
High SS vs Moderate SS among opioid users	0.41	0.23 – 0.72	0.002
High SS vs Moderate SS among non-opioid users	0.50	0.35 – 0.71	< 0.0001
**Rural high school**			
High SS vs Low SS among opioid users	0.44	0.20– 0.96	0.040
High SS vs Low SS among non-opioid users	0.33	0.21 – 0.51	< 0.0001
Moderate SS vs Low SS among opioid users	0.70	0.38 – 1.30	0.263
Moderate SS vs Low SS among non-opioid users	0.66	0.42 – 1.03	0.067
High SS vs Moderate SS among opioid users	0.62	0.34 – 1.14	0.125
High SS vs Moderate SS among non-opioid users	0.49	0.32 – 0.76	0.001
**Urban high school**			
High SS vs Low SS among opioid users	0.46	0.18 – 1.22	0.119
High SS vs Low SS among non-opioid users	0.48	0.27– 0.86	0.013
Moderate SS vs Low SS among opioid users	1.62	0.64 – 4.13	0.303
Moderate SS vs Low SS among non-opioid users	0.81	0.45 – 1.46	0.487
High SS vs Moderate SS among opioid users	0.28	0.12 – 0.66	0.004
High SS vs Moderate SS among non-opioid users	0.59	0.38 – 0.93	0.024
**Rural Off Reservation**			
High SS vs Low SS among opioid users	0.39	0.16 – 0.93	0.035
High SS vs Low SS among non-opioid users	0.32	0.18 – 0.55	< 0.0001
Moderate SS vs Low SS among opioid users	0.80	0.41 – 1.57	0.512
Moderate SS vs Low SS among Non-opioid users	0.74	0.43 – 1.24	0.252
High SS vs Moderate SS among opioid users	0.48	0.24 – 0.99	0.047
High SS vs Moderate SS among non-opioid users	0.43	0.26 – 0.71	0.001
**Rural On Reservation**			
High SS vs Low SS among opioid users	0.63	0.12 – 3.45	0.589
High SS vs Low SS among non-opioid users	0.37	0.20 – 0.68	0.002
Moderate SS vs Low SS among opioid users	0.31	0.07 – 1.38	0.121
Moderate SS vs Low SS among non-opioid users	0.41	0.20 – 0.86	0.019
High SS vs Moderate SS among opioid users	2.05	0.75 – 5.55	0.155
High SS vs Moderate SS among non-opioid users	0.90	0.52 – 1.56	0.708
**Urban Off Reservation**			
High SS vs Low SS among opioid users	0.47	0.18 – 1.22	0.119
High SS vs Low SS among non-opioid users	0.48	0.27 – 0.86	0.013
Moderate SS vs Low SS among opioid users	1.63	0.64 – 4.13	0.303
Moderate SS vs Low SS among non-opioid users	0.81	0.45 – 1.46	0.487
High SS vs Moderate SS among opioid users	0.29	0.12 – 0.67	0.004
High SS vs Moderate SS among non-opioid users	0.59	0.38 – 0.93	0.024

### Reservation and rurality

Students who reported high social support and opioid misuse and attended school off-reservation had a significantly lower odds for suicide attempt (AOR = 0.42; 95% CI: 0.21 – 0.82; *p* = 0.012) relative to those who reported low social support. A similar significantly lower odds ratio for suicide attempt was found for students who reported high social support and no opioid misuse and attended school on-reservation (AOR = 0.37, 95% CI: 0.20 – 0.68; *p* = 0.002) compared to those with low social support, and students who reported high social support and no opioid misuse (AOR = 0.37; 95% CI: 0.25 – 0.56; *p* < 0.0001) compared to those with low social support (Table 3). Students reporting moderate social support and no opioid misuse and attended on-reservation schools had a lower odds ratio for suicide attempt (AOR = 0.41; 95% CI: 0.20-0.86, *p* = 0.019) relative to students with low social support. For students who attend off-reservation high schools, those who reported high social support and opioid misuse had a significantly lower odds ratio for suicide attempt (AOR = 0.41; 95% CI: 0.23-0.72, *p* = 0.002) compared to those with moderate social support; whereas students who did not report opioid misuse had an odds ratio of 0.50 (95% CI: 0.35-0.71, *p* < 0.0001) for suicide attempt.

Among those attending schools on-reservation, no significant associations were found for students who reported high social support and opioid misuse (AOR = 0.63, 95% CI: 0.12-3.45, *p* = 0.589) relative to those with low social support, students reporting moderate social support and opioid misuse (AOR = 0.31, 95% CI: 0.07-1.38, *p* = 0.121) compared to those reporting low social support, students who reported high social support and opioid misuse (AOR = 2.05, 95% CI: 0.75-5.55, *p* = 0.155) compared to those with moderate social support, and students who reported high social support and no opioid misuse (AOR = 0.90, 95% CI: 0.52-1.56, *p* = 0.708) compared to those with moderate social support. For students who attend off-reservation schools, we did not find significant associations for students who reported moderate social support and either misused opioids (AOR = 1.02, 955 CI: 0.59-1.78, *p* = 0.943), or did not misuse opioids (AOR = 0.75, 95% CI:0.50-1.12, *p* = 0.154) compared to those with low social support.

In rural high schools, students who reported high social support and opioid misuse had a significantly lower odds ratio for suicide attempt (AOR = 0.44, 95% CI: 0.20-0.96, *p* = 0.040), and those who did not report opioid misuse also had a significantly lower odds ratio for suicide attempts (AOR = 0.00, 95% CI: 0.21-0.51, *p* < 0.0001) compared to those with low social support. Students who reported high social support and no opioid misuse had a significantly lower odds ratio for suicide attempt (AOR = 0.49, 95% CI: 0.32-0.76, *p* = 0.001) relative to those with moderate social support. In urban high schools, students who reported high social support and no opioid misuse had an odds ratio of 0.48 (95% CI: 0.27-0.86, *p* = 0.013) for suicide attempt compared to those with low social support. Comparing high social support and moderate social support, students who reported opioid misuse had a significantly lower odds for suicide attempt (AOR = 0.28, 95% CI: 0.12-0.66, *p* = 0.004), and students who did not report opioid misuse had a significantly lower odds of suicide attempt (AOR = 0.59, 95% CI: 0.38-0.93, *p* = 0.024).

No significant associations were found for students in rural high schools who reported moderate social support and either opioid misuse or not compared to low social support, and students who reported high social support and opioid misuse compared to moderate social support (Table 3). For students in urban high schools, we also found no significant associations for students who reported high social support and opioid misuse compared to low social support, students who reported moderate social support and either opioid misuse or not compared to those with low social support (Table 3).

### Rural off reservation, rural on reservation, and urban off reservation

Students in rural off-reservation schools who reported high social support and either opioid misuse (AOR = 0.39, 95% CI: 0.19-0.93, *p* = 0.035) or no opioid misuse also had a significantly lower odds ratio for suicide attempt (AOR = 0.32, 95% CI: 0.18-0.55, *p* < 0.001) compared to those with low social support. In addition, a borderline significantly lower odds for suicide attempt (AOR = 0.48, 95% CI: 0.24-0.99, *p* = 0.047) was found for students who reported high social support and opioid misuse compared to those with moderate social support. Students who reported high social support and no opioid misuse had a significantly lower odds ratio of 0.43 for suicide attempt (95% CI: 0.26-0.71, *p* = 0.001) compared to those with moderate social support. In rural on-reservation schools, students who reported high social support and no opioid misuse had a significantly lower odds ratio for suicide attempt (AOR = 0.37, 95% CI: 0.20-0.68, *p* = 0.002) compared to those with low social support. Likewise, students who reported moderate social support and no opioid misuse also had a significantly lower odds ratio for suicide attempts (AOR = 0.41, 95% CI: 0.20-0.86, *p* = 0.019) relative to those with low social support. For students in urban off-reservation schools, those who reported high social support and no opioid misuse had a significantly lower odds ratio for suicide attempt (AOR = 0.48, 95% CI: 0.27-0.86, *p* = 0.013) compared to those with low social support. Comparing high and moderate social support, students who reported opioid misuse and no opioid misuse had a significantly lower odds for suicide attempt (AOR = 0.29, 95% CI: 0.12-0.67, *p* = 0.004; AOR = 0.59, 95% CI: 0.38-0.93, *p* = 0.024, respectively).

Comparing moderate social support and low social support for students at rural off- reservation schools, no significant associations were found for students who reported either opioid misuse or no opioid misuse (Table 3). For rural schools on reservations, no significant association was found for students who reported high social support and opioid misuse compared to those with low social support. Comparing moderate social support and low social support, no significant association for suicide attempt was found for students who reported opioid misuse. There were no significant associations for students who reported high social support and opioid misuse, and students who reported high social support and no opioid misuse relative to those who reported moderate social support (Table 3). In urban schools off-reservation, no significant association was found for students who reported high social support and opioid misuse relative to low social support. We also found no significant associations when comparing moderate social support and low social support among students who reported opioid misuse and no opioid misuse (Table 3).

### Mediation analysis

Overall, the controlled direct effect (CDE) of opioid misuse on suicide attempt, in the absence of social support was 53.77% (95% CI: 51.01-56.54; *p* < 0.0001). About 24 % (23.64%) of the association between opioid misuse and suicide attempt (PIE: pure indirect effect) was mediated by social support (95% CI: 21.68 – 25.60; *p* < 0.0001), and about 41.05% of the association was due to the moderation effect of social support and opioid misuse (95% CI: 38.64 – 43.46; *p* < 0.0001) (Table 4).

**Table 4:** Mediation Analysis for social support on the association between opioid misuse and suicide attempt

Residency	Opioid Misuse	Social Support	Prevalence of Suicide Attempt (%)	Controlled Direct Effect (CDE): Due Neither to Mediation nor Interaction	Reference Interaction (INT_**ref**_): Due to Interaction Only	Mediated Interaction (INT_**med**_): Due to Mediation and Interaction	Pure Indirect Effect (PIE): Due to Mediation Only
				Percent (95% CI) *P*-value	Percent (95% CI) ***P***-value	Percent (95% CI) *P*-value	Percent (95% CI) *P*-value
Overall	Yes	low SS	50.12	53.77 (51.01-56.54) *p* < 0.0001	41.05 (38.64-43.46) *p* < 0.0001	−18.46 (−19.97--16.95) *p* < 0.0001	23.64 (21.68-25.60) *p* < 0.0001
		Moderate SS	38.65				
		High SS	23.42				
	No	low SS	18.04				
		Moderate SS	11.45				
		High SS	7.42				
Rural on reservation	Yes	low SS	49.1	72.38 (66.14-78.63) *p* < 0.0001	24.12 (18.69-29.56) *p* < 0.0001	−13.20 (−16.56- -9.83) *p* < 0.0001	16.69 (12.38-21.00) *p* < 0.0001
		Moderate SS	34.01				
		High SS	30.25				
	No	low SS	17.31				
		Moderate SS	10.37				
		High SS	7.73				
Rural off reservation	Yes	low SS	51.44	43.43 (39.83-47.02) *p* < 0.0001	49.52 (46.47-52.57) *p* < 0.0001	−21.25 (−23.43- -19.06) *p* < 0.0001	28.30 (25.33-31.27) *p* < 0.0001
		Moderate SS	35.14				
		High SS	20.93				
	No	low SS	20.01				
		Moderate SS	12.53				
		High SS	6.55				
Urban off reservation	Yes	low SS	46.79	60.36 (54.50-66.21) *p* < 0.0001	36.30 (30.95-41.66) *p* < 0.0001	−15.88 (−18.68- -13.08) *p* < 0.0001	19.22 (15.79-22.65) *p* < 0.0001
		Moderate SS	46.5				
		High SS	25.66				
	No	low SS	15.6				
		Moderate SS	9.5				
		High SS	8.17				

However, restricting the analysis to AI/AN youth who attend rural on-reservation schools, the CDE of opioid misuse on suicide attempt, with no social support was 72.38% (95% CI: 66.14 – 78.63; *p* < 0.0001). The interaction effect of opioid misuse and social support made up 24.12% (95% CI: 18.69 – 29.56; *p* < 0.0001) of the total association with suicide attempt whereas mediation made up only 16.69% (95% CI: 12.38 – 21.00; *p* < 0.0001) of the total association (Table 4).

For students who attended rural off-reservations schools, the effects of opioid misuse on suicide attempt in the absence of social support (CDE) was 43.43% (95% CI: 39.83 – 47.02; *p* < 0.0001). The interaction effect of opioid misuse and social support contributed about 49.52% (95% CI: 46.47 – 52.57; *p* < 0.0001) of the total association with suicide attempt whereas the mediation through social support contributed 28.30% (95% CI: 25.33 – 31.27; *p* < 0.0001).

For students who attended urban off-reservation high schools, the effects of opioid misuse on suicide attempt (CDE) were 60.36% (95% CI: 54.50 – 66.21; *p* < 0.0001); however, the interaction effect of opioid misuse and social support made up 36.30% (95% CI: 30.95 – 35.66; *p* < 0.0001) of the total association with suicide attempt (Table 4). The pure indirect effect (PIE) of social support on the association between opioid misuse and suicide attempt was 19.22% (95% CI: 15.79 – 22.65; *p* < 0.0001).

## Discussion

The primary objective of this study was to examine how social support mediates the association between opioid misuse and suicide attempt among AI/AN youth in New Mexico. We found that high social support is broadly protective for suicide attempt, as it is associated with reduction in this risk among AI/AN youth who misuse opioids, as well as those who do not. But the risk reduction was greater among those who did not misuse opioids. This finding is consistent with prior research [33, 35, 46, 51, 56]. Strong familial support, cultural ties, and other social support, such as school personnel have been identified as significant resilience factors in AI/AN communities [18, 27, 46, 51, 53, 57], and some tribes are leveraging these areas as alternatives to interventions that only use clinical and behavioral therapy and to mitigate behavioral health risk [17, 33, 50]. This strengths-based analysis highlights how social connections with family, teachers, friends, and other mentors are key resources in AI/AN communities that improve youth health and should be integrated into behavioral health initiatives targeting youth suicide and substance use interventions.

Whereas our study revealed relatively lower mediation effect of social support on the association between opioid misuse and suicide attempt, the moderation effect of social support and opioid misuse on suicide attempt was relatively more pronounced. The mediation role of social support in the association between opioid misuse and suicide attempt was more profound among AI/AN male youth compared to females. While there was no significant sex difference in opioid misuse among New Mexico AI/AN students in these data, our analysis suggests that social support appears to have different effects. Possible explanations are gender role differences within AI/AN communities in New Mexico. In some communities, male youth may have significant expectations to learn and perpetuate cultural values and traditions and to take up leadership roles. As such, male AI/AN youth may especially benefit from social support as a way to manage community expectations as they learn to fulfill tribal gender roles [58].

Moreover, our study revealed that high social support compared to low social support was more impactful among AI/AN youth who attended schools off-reservation in reducing the risk of suicide attempt for students who misused opioids. Compared to students attending schools in reservation communities, social support had a greater impact on AI/AN youth attending schools outside of the reservation, which may be because they might have adopted some other cultural experiences in addition to the native culture. There is evidence that AI/AN adolescents who have bicultural competence have significantly less hopelessness feelings [59]. More research is needed in this area. In addition, attending off-reservation schools might also grant students access to other healthcare services and other social support systems which might not be available in on-reservation schools. This is more reflective in a study that measured the academic achievement of American Indians and identified some gaps in the academic performance of AI/AN on-reservation compared to those off-reservation [60]. Furthermore, there are more tribal specific dimensions of social support like clan relationships and participating in tribal rituals that might not have been captured using the survey instrument.

Students attending -off-reservation rural schools also demonstrated greater benefit from high social support on the relationship between opioid misuse and suicide attempt than those who attended urban schools. This may be due in part to trends shown in previous research suggesting students who attend urban schools and have high social support still have higher odds of suicide attempt than their counterparts who attend rural schools [27]. While enhancing social support in urban off reservation schools is needed, the highest attention should be for rural on reservation schools.

Increasing social support is critical to improving AI/AN youth behavioral health challenges. Parents or guardians of AI/AN youth should be heavily involved in the day -to-day activities of the youth under their care. This may include engagement with their academic, recreational, and other social activities, which help to create strong positive relationships. Such positive familial environments mediate the negative impact of suicide attempts, and opioid misuse [33, 51]. Schools could also play a critical role in mitigating the risk for suicide attempts and opioid misuse by establishing teen health centers in schools with an on-site mental health counselor, and incorporating behavioral health skills development into the curricula [5], such as teaching students first aid skills for suicide prevention through role-play and group discussions [61]. At the community level, AI/AN elders could collaborate with schools to visit classrooms and help youth connect with their culture, traditions and heritage through elder taught lessons [62], and also connect students to tribal ceremonies [63]. It is imperative that cultural awareness be incorporated into social support programs. Local service providers could coordinate their efforts in data collection and combining local data repository to form a larger dataset across AI/AN communities. This approach would help augment evidence-based research for culturally based suicide prevention and other behavioral health programs [64].

Past studies have highlighted the importance of different levels of social support in adolescent behavioral health [43, 65–67]. Interpersonal levels like individual self-esteem or self-efficacy, positive mood or good emotional health protect AI/AN youth from suicide attempts and other related behavioral health issues [49, 66]. In addition, attention should also be given to other levels of social support such as the societal, and community levels [35]. Exploring the intersection of multiple levels of social support for AI/AN youth has the potential to strengthen intervention programs in ways that not only prevent deaths, but also create environments where youth can thrive [43, 67].

This study has limitations related to the data used for the analysis. First, the study relied on self-reported information and may be subject to recall bias. The self-reported data are not able to measure students who died by suicide, only those who attempted suicide without completing. Second, due to the cross-sectional design of the data, inference about the temporality of the association is not possible; hence we do not imply causation. These data only include students enrolled in school. By 12th grade, youth who were misusing opioids may have dropped out or been expelled from school. In addition, the survey measurements were not validated for AI/AN youth and may not account for important cultural considerations in how social support is perceived and valued. To the best of our knowledge, this is the first study to examine the mediation and moderation effects of social support on the association between opioid misuse and suicide attempt among AI/AN youth in New Mexico. This is a key contribution to behavioral health research in AI/AN communities in the Southwest because it highlights the role community members play in mitigating risk. While many studies highlight the risk for substance use and suicide among AI/AN populations, this study provides estimates of the significant role community resiliency factors play in promoting health.

Future studies should explore the use of culturally validated instruments for measuring substance use, suicide attempts and social support. Moreover, survey respondents were not able to be matched across waves of data collection. A longitudinal study to measure the effectiveness of social support as a mediator of the association between opioid misuse and suicide attempt is highly needed.

## Conclusion

Social support mediates the association between opioid misuse, and suicide attempt. High social support relative to low social support was associated with the reduction in the risk of suicide attempt among males, and females who misuse opioids. Among students who misused opioids, high social support relative to low social support was associated with the reduction in the risk of suicide attempt for students who attended high school in off reservation, and rural communities. The mediation effects of social support on the association between opioid misuse and suicide was least among students in rural on-reservation communities. More resources need to be allocated to rural on-reservation communities to enhance social support programs.

## Data Availability

The datasets generated and/or analyzed during the current study are not publicly available due to restrictions by the New Mexico Department of Health (NMDOH), and the Albuquerque Area Southwest Tribal Epidemiology Center (AASTEC); the providers of the AI/AN oversampled NMYRRS data. Data can be accessed by signing a data sharing agreement with NMDOH and AASTEC (Dr. Kevin English: https://www.aastec.net/; kenglish@aaihb.org).
