# Correction: The mediation and moderation effect of social support on the relationship between opioid misuse and suicide attempts among native American youth in New Mexico: 2009-2019 Youth Risk Resiliency Survey (NM-YRRS)

**DOI:** 10.1186/s12888-022-03993-1

**Published:** 2022-06-09

**Authors:** Daniel Opoku Agyemang, Erin Fanning Madden, Kevin English, Kamilla L. Venner, Rod Handy, Tejinder Pal Singh, Fares Qeadan

**Affiliations:** 1 Department of Family and Preventive Medicine, University of Utah School of Medicine, Salt Lake City, USA; 2 Department of Family Medicine and Public Health Sciences, Wayne State University, Detroit, USA; 3 Albuquerque Area Southwest Tribal Epidemiology Center, Santa Fe, NM USA; 4 Department of Psychology, Center on Alcohol, Substance use, And Addiction (CASAA), University of New Mexico, Albuquerque, USA; 5 Parkinson School of Health Sciences and Public Health, Loyola University Chicago, Maywood, IL, US United States

**Correction: BMC Psychiatry 22, 243 (2022)**

**https://doi.org/10.1186/s12888-022-03900-8**

Following publication of the original article [1], the authors identified that the footnote was placed in the Results section. The footnote is placed below Table 2.

**Table 2:** Characteristics of AI/AN youth participants (New Mexico YRRS 2009-2019)

	Total n (%^**1**^)	AI/AN Youth with Opioid Misuse n (%^**2**^)	AI/AN Youth with no Opioid Misuse n (%^**2**^)	***P***-value
Total	19,067 (100%)	2,363 (12.0)	16,704 (88.0)	NA
**Mediating variable**				
Social Support (SS)				
Low SS	3,258 (17.5)	720 (22.0)	2,538 (78.0)	<0.0001
Moderate SS	5,945 (31.6)	874 (14.5)	5,071 (85.5)	
High SS	9,864 (50.9)	769 (7.3)	9,095 (92.7)	
**Control variables**				
Age (years)				
≤14	3,826 (20.8)	361 (8.7)	3,465 (91.3)	0.0001
15	4,936 (26.1)	612 (12.8)	4,324 (87.2)	
16	4,899 (24.5)	640 (12.9)	4,259 (87.1)	
≥17	5,406 (28.6)	750 (13.4)	4,656 (86.6)	
Sex				
Female	9,807 (49.4)	1202 (11.8)	8,605 (88.2)	0.3870
Male	9,260 (50.6)	1,161 (12.5)	8,099 (87.5)	
Grade				
9th	5,626 (29.9)	614 (9.8)	5,012 (90.2)	0.0039
10th	5,053 (26.7)	631 (13.2)	4,422 (86.8)	
11th	4,511 (23.0)	618 (13.3)	3,893 (86.7)	
12th	3,762 (20.4)	473 (11.9)	3,289 (88.1)	
Academic performance				
High grades (A's / B's)	11,577 (69.0)	1,106 (9.5)	10,471 (90.5)	<0.0001
Poor grades (C, D, or F's)	5,143 (31.0)	902 (17.0)	4,241 (83.0)	
Maternal education level				
<High School	2,349 (18.0)	352 (15.6)	1,997 (84.4)	0.0001
High School	7,534 (50.0)	864 (10.9)	6,670 (89.1)	
≥College	4,437 (32.0)	459 (10.0)	3,978 (90.0)	
Sexual identity				
Heterosexual	10,633 (81.1)	869 (8.1)	9,764 (91.9)	<0.0001
Gay/Lesbian	353 (2.9)	107 (29.9)	246 (70.1)	
Bisexual	1,493 (11.4)	315 (22.1)	1,178 (77.9)	
Questioning	547 (4.6)	96 (22.4)	451 (77.6)	
**Stratifying variables**				
Reservation status of school				
On	4066 (13.0)	484 (10.0)	3,582 (90.0)	0.041
Off	14,108 (87.0)	1,767 (12.0)	12,341 (88.0)	
Rurality status of school				
Rural	13,782 (66.0)	1,727 (13.0)	12,055 (87.0)	0.187
Urban	4,392 (34.0)	524 (11.0)	3,868 (89.0)	

1 %=column percentage

2 %=row percentage

The original article [1] has been corrected.
